# Validity of Actigraphy Compared to Polysomnography for Sleep Assessment in Children With Autism Spectrum Disorder

**DOI:** 10.3389/fpsyt.2019.00551

**Published:** 2019-08-02

**Authors:** Enise Yavuz-Kodat, Eve Reynaud, Marie-Maude Geoffray, Nadège Limousin, Patricia Franco, Patrice Bourgin, Carmen M. Schroder

**Affiliations:** ^1^Centre National de la Recherche Scientifique, Université de Strasbourg, Institut des Neurosciences Cellulaires et Intégratives, Strasbourg, France; ^2^Department of Child and Adolescent Neurodevelopmental Psychiatry, Le Vinatier Hospital, Bron, France; ^3^Health Services and Performance Research (HESPER), Claude Bernard University Lyon 1, Lyon, France; ^4^Department of Neurology and Clinical Neurophysiology, University Hospital Bretonneau, Tours, France; ^5^Lyon Neuroscience Research Center U1028/UMR 5292, Claude Bernard University Lyon 1, Lyon, France; ^6^Sleep Disorders Center, International Research Center for ChronoSomnology, Strasbourg University Hospitals, Strasbourg, France; ^7^Department of Child and Adolescent Psychiatry, Strasbourg University Hospitals & University of Strasbourg Medical School, Strasbourg, France

**Keywords:** autism, autism spectrum disorder, actigraphy, actimetry, polysomnography, PSG, validation, sleep

## Abstract

Actigraphy (ACT) is a non-invasive objective assessment tool for the study of sleep–wake rhythms. It is of particular interest in children with autism spectrum disorder (ASD), as sleep disorders are highly prevalent and have a significant impact on both cognitive and behavioral functions. As polysomnography (PSG), the gold standard for the assessment of sleep, is difficult to perform in children with ASD, ACT has become a tool of choice but has not yet been validated against PSG using state-of-the-art methodology. The main objective of this study was to assess, for the first time, the validity of ACT compared to PSG for the measurement of sleep in children with ASD. During the same night of hospitalization, PSG and ACT were conducted in 26 children (6 girls and 20 boys; mean age 5.4 years ± 1.6) diagnosed with ASD according to DSM-5 criteria and standardized diagnostic scales. Sleep parameters were total sleep time (TST), sleep latency (SL), wake after sleep onset (WASO), and sleep efficiency (SE). To compare PSG and ACT, we conducted sleep parameter agreement analyses including: intraclass correlation coefficient (ICC), Bland-Altman plots, and equivalence tests. The comparison also included an epoch-by-epoch (EBE) agreement analysis to determine sensitivity (ability to detect sleep) and specificity (ability to detect wake). According to equivalence tests, the difference between ACT and PSG measures was clinically acceptable for TST (<30 min, p < 0.01), SL (<15 min, p < 0.001), and SE (10%, p < 0.01), but not for WASO (<15 min, p = 0.13). There was a good agreement between methods for SL (ICC = 0.79) and TST (ICC = 0.85) and a moderate agreement for WASO (ICC = 0.73) and SE (ICC = 0.68). The EBE agreement analysis revealed a high sensitivity (0.94 ± 0.06) and moderate specificity (0.5 ± 0.2). Since sleep disorders are one of the most common comorbidities within the ASD population and are highly prevalent, it is essential to validate objective tools of assessment. To our knowledge, our study is the first to validate ACT compared to PSG, using a state-of-the-art methodology, in children with ASD. The results suggest ACT to be a valid method to evaluate sleep within this population, with a good reliability for most sleep parameters.

## Introduction

Autism spectrum disorder (ASD) is a neurodevelopmental disorder characterized by a persistent impairment in reciprocal social communication and social interaction, and restricted, repetitive patterns of behavior, interests, or activities. Symptoms are present from early childhood and significantly affect daily functioning (1). ASD is four times more common in males than in females, and its worldwide reported prevalence approaches 1% of the population (2–4). Comorbidities are very common in ASD. Indeed, over 70% of individuals diagnosed with ASD have concurrent somatic or psychiatric conditions (5, 6). Sleep disorders are among the most common associated disorders in this population, with prevalence rates ranging from 50 to 80% (7, 8).

Studies based on parent-reported sleep problems have shown that the most common complaints are related to bedtime resistance, sleep initiation, nighttime awakening, and shortened sleep time (9). In accordance with these findings, studies using objective measurements of sleep quality parameters have revealed that children with ASD, compared to typically developing children (TD), display increased sleep onset latency, decreased sleep efficiency, as well as an increased number and duration of night wakings (10, 11). Research also provides support for the idea that sleep disturbances are strongly associated with daytime functioning in children with ASD. In a recent study, Mazurek and Sohl (12) showed that sleep disturbances significantly account for behavioral dysregulation, notably inattention, impulsivity, irritability, and physical aggression (12). They also showed that night wakings have the most consistently strong association with daytime behavior problems. Other authors found that sleep disturbances are significantly correlated with both internalizing and externalizing symptoms in children with ASD, using the Pediatric Behavior Scale (13). Studies have further suggested that ASD children who experience sleep problems have also cognitive impairment. For example, decreased sleep duration in children with ASD was correlated with nonverbal communication deficits (14), lower overall intelligence, adaptive functioning, and socialization skills (15, 16).

Finally, it is well characterized that sleep disturbances worsen quality of life of both children with ASD and their families (17, 18). Levin and Scher reported that sleep problems contributed significantly to maternal stress (19).

If not treated early, sleep disorders persist from infancy to adolescence (20). Thus, it is essential to address sleep problems in children with ASD, in order to favorably impact not only nocturnal symptoms, but also their daily functioning as well as overall quality of life of these children with ASD and those of their caregivers.

In order to efficiently design sleep interventions and overall medical care, it is essential to assess sleep quality parameters in children with ASD. Polysomnography (PSG) is the gold standard for sleep quality assessment but, aside from the cost involved and its limited availability, it can be challenging and often impossible to conduct PSG in this population (21). Indeed, polysomnographic recording may be compromised because many children with ASD present sensory abnormalities, and thus may not tolerate electrodes on their scalp or face (11). As an alternative, actigraphy (ACT) has been used as a non-invasive, objective, and cost-effective assessment tool for the study of rest-activity cycles as a proxy to sleep–wake rhythms. It has become a tool of choice to assess sleep quality in children with ASD. However, to the best of our knowledge, no study has yet investigated the validity of ACT compared to PSG for the measurement of sleep in children with ASD.

The aim of this study was to compare the agreement of actigraphy (MotionWare 8®—CamNtech MotionWare 1.1.20) with gold standard polysomnography in children diagnosed with ASD.

## Materials and Methods

### Participants

Participants were recruited as part of a French multicenter clinical research program (university hospitals of Strasbourg, Lyon, and Tours), examining the role of sleep disorders and circadian rhythm disorders in children with ASD.

The study complied with the principles of the Declaration of Helsinki (1989) and standards of good clinical practices. All procedures have been approved by the regional French Institutional Review Board (Comité de Protection des Personnes “Est IV”, 11/04/2012, 1 place de l’hôpital 67091, Strasbourg). Written, signed, and informed consent was obtained prior to participation from the parents of participants, and assent was obtained from the child when possible.

Inclusion criteria were a diagnosis of ASD using the *Diagnostic and Statistical Manual of Mental Disorders* (DSM) IV-R/5 criteria (1). All children underwent an initial diagnostic evaluation including the Autism Diagnostic Observation Scale [ADOS (22)], and the Autism Diagnostic Interview-Revised [ADI-R (23)] which was completed with parents. All participants met diagnostic criteria for ASD using the ADOS cutoff and met criteria on all domains of the ADI-R. The ADOS and ADI are gold standard measures for the diagnosis of ASD and were administered by certified practitioners. Furthermore, children had to be on stable medication 2 months before the inclusion and during the assessment periods.

Exclusion criteria were secondary ASD (e.g., associated with fragile X syndrome, Rett syndrome, Down syndrome, Bourneville tuberous sclerosis, Von Recklinghausen’s disease, cytomegalovirus encephalitis, congenital rubella syndrome, and phenylketonuria). Patients with epilepsy, comorbid severe physical disability, or severe allergy were also excluded from the study. Participants were not allowed to have had transmeridian travels over two time zones or more, 3 months before the assessments.

Twenty-nine children with ASD were included into this study and underwent a night of polysomnography assessment as part of the overall research protocol, while wearing concomitantly an actigraphy wrist watch.

### Measures

Sleep quality parameters used in this study were: TST, SL, WASO, and SE; TST was defined as the time between sleep onset and sleep offset minus the time of WASO, and SL was defined as the time between bedtime and sleep onset. WASO was defined as the number of minutes scored as wake between sleep onset and sleep offset. SE was defined as the ratio of TST to the time in bed (i.e., time from bedtime to get up time). We defined “bedtime” as the moment when the child is in bed, ready to sleep, and “get up time” as the moment when the child is getting up.

#### Actigraphy

Children wore an actigraph (the MotionWare 8®—CamNtech MotionWare 1.1.20) on their non-dominant wrist or on the left wrist by default if the child was not lateralized yet. The actigraph is an electronic device containing a piezo-electric accelerometer that measures the intensity, the amount, and the duration of physical movement in all directions. The actigraph activity is measured in counts defined as the amplitude of the signal produced by the accelerometer in the actigraph, with the number of counts being proportional to the intensity of the movement. The accelerometer samples the amplitude of the movement 32 times per second. The peak of intensity is defined for each second as the highest amplitude within the 32 records, and peak intensity values are summed into an epoch of 1 min.

Actigraphy data were scored automatically for sleep/wake using the Actiwatch Activity and Sleep Analysis 7® software algorithm, version 7.23. We used four different sensitivity-threshold settings: automatic, low, medium, and high. The low sensitivity-threshold is defined as a threshold level of 80 counts, i.e., an activity score of 80 counts or more during one epoch is necessary for that epoch to be scored as wake. The medium sensitivity threshold corresponds to a threshold level of 40 counts, the high sensitivity threshold to a threshold level of 20 counts, and the automatic sensitivity threshold to a variable threshold level derived from the subject’s individual activity level. The low sensitivity-threshold setting of actigraphy showed the best fit for the comparison of actigraphy-derived sleep parameters to PSG and was thus reported here (for all other settings, please refer to **Supplementary Data**).

While activity counts were recorded in 1-min epochs, they were converted into 30-s epochs for the epoch-by-epoch analyses, as done in previous studies (24, 25), in order to allow for comparison with PSG data. The conversion has been done using the provided feature in the actigraphy reading software (MotionWare 8®), which consists in dividing a 1-min epoch of wake in two 30-s epochs of wake and similarly so for the sleep epochs.

#### Polysomnography

Polysomnography was conducted with Compumedics Siesta 802a (Compumedics, Abbotsford, Australia). We collected the following channels for sleep staging: 13 electroencephalogram (EEG) channels (FP1, FP2, F3, F4, C3, Cz, C4, T3, T4, M1(A1), M2(A2), O1, O2), bilateral electrodes for electrooculogram (E2, E1) and submental electromyogram (Chin2–Chin1), and electrocardiograms (ECG+, ECG-) and sensors to monitor airflow. Raw data were digitalized at a sampling rate of 1,024 Hz, and a notch filter of 50 Hz was applied. PSG studies were manually double scored by two independent raters using Compumedics Profusion PSG V4.1 version 445 Software following the American Academy of Sleep Medicine (AASM) guidelines for sleep staging in 30-s epochs (26).

### Data Analyses

The comparison of polysomnography- and actigraphy-derived sleep parameters was based on a single concurrent night of recording and included two sets of comparisons as primary analyses: 1) the agreement analysis of the four sleep parameters: TST, SOL, WASO, and SE, and 2) an epoch-by-epoch agreement analysis. For secondary analyses, we compared the ACT sensitivity-threshold settings for each sleep parameter and epoch-by-epoch variables, as reported in the **Supplementary Data**. All analyses were restricted to nighttime sleep–wake patterns.

All statistical analyses were performed using R Statistics Software Version 3.4.3.

#### Sleep Parameter Agreement Analyses

For the sleep parameter analysis, we performed a state-of-the-art agreement analysis method (27) using ICC, Bland-Altman plots, and Yuen two one-sided paired equivalence test (28).

The ICC is an index that, contrary to Pearson correlation, assesses not only how well correlated the two techniques are but also if they are equal. ICC ranges from 0 (no agreement) to 1 (perfect agreement). An ICC < 0.5 indicates poor agreement, 0.5 < ICC > 0.75 indicates moderate agreement, 0.75 < ICC > 0.9 indicates good agreement, and ICC > 0.90 indicates perfect agreement (29).

Bland-Altman plots are a graphical method that allows to visually examine the degree of agreement between two techniques. In this method, the differences between PSG and ACT (i.e., SL according to actigraphy minus SL according to PSG) are plotted against their averages. The plot includes one value for each subject, a reference line (equal to zero, representing perfect agreement between PSG and ACT), the mean of the differences between the two techniques (representing the mean bias), and limits of agreement (which are defined as a deviation from the mean superior to two standard deviations).

Finally, the Yuen two one-sided paired tests for equivalence allow to conclude if two techniques are *clinically* equivalent within a pre-set range of acceptability. In equivalence tests, the null and alternative hypotheses are reversed compared to usual tests (e.g., Student t-test). The null hypothesis of an equivalence test states that there is a difference between conditions, whereas the alternate hypothesis states that there is no difference (28). This test is necessary in order to attest a true clinical equivalence between two tests, when the null hypothesis is rejected (p-value < 0.05). In our study, the ranges were set to ±30 min for TST, ±15 min for SL, and ±15 min for WASO. We set two ranges for SE, a conservative one to ±5%, and an extended one to ±10%.

#### Epoch-by-Epoch Agreement Analysis

Each epoch, comprised of 30 s of recording, was coded as a binary score (W = wakefulness and S = sleep) for both ACT and PSG.

PSG being the gold standard, coding by this method was defined as the accurate state. As detailed in **Table 1**, epochs where ACT accurately identified sleep or wake were respectively called true sleep (TS) and true wake (TW). Conversely, epochs where ACT misidentified sleep for wake were called false wake (FW), and those where ACT misidentified wake for sleep were called false sleep (FS).

**Table 1:** Definition of epoch qualification for the epoch-to-epoch agreement analysis.

	PSG	
			Sleep	Wake
ACT	Sleep	True sleep (TS)	False sleep (FS)
	Wake	False wake (FW)	True wake (TW)

Epoch-by-epoch analysis consisted in calculating accuracy, sensitivity, and specificity for all of the sensitivity settings (automatic, high, medium, and low). Accuracy was defined as the number of epochs that ACT correctly classified into sleep or wake (as defined by PSG) divided by the total number of epochs: *(TS + TW)/(TS + TW + FS + FW)*. Sensitivity was calculated as the number of epochs where ACT correctly identified sleep, divided by the number of epochs scored as sleep by PSG: *TS/(TS+FW)*. Specificity was defined as the number of epochs correctly identified as wake by ACT, divided by the number of epochs scored as wake by PSG: *TW/(TW+FS)*. Sensitivity answers the question, “What percentage of PSG sleep epochs are detected by ACT?”, and specificity answers to the question “What percentage of PSG wake epochs are detected by ACT?” (30).

We also computed the predicted value for sleep (PVS), which is the percentage of epochs scored as sleep by ACT that were also scored as sleep by PSG: *TS/(TS+FS)*, and the predicted value for wakefulness (PVW), which is the percentage of epochs scored as wake by ACT that were also scored as wake by PSG: *TW/(TW+FW)* (31). PVS answers the question, “Within epochs identified as sleep by ACT what is the percentage of PSG sleep?,” and PVW answers the question “Within epochs identified as wake by ACT, what is the percentage of PSG wake?” We also calculated the Cohen’s kappa coefficient value (k) for all sensitivity settings of the actigraph. The k coefficient is an indicator that reflects the percentage of scoring agreement between two techniques (PSG and ACT) which is not due to chance (32). We considered a kappa coefficient of 0–0.2 as slight agreement, 0.2–0.4 as fair agreement, 0.4–0.6 as moderate agreement, 0.6–0.8 as substantial agreement, and 0.8–1.0 almost perfect agreement (33).

#### ACT Sensitivity-Threshold Setting Analysis

ANOVA was used to compare the sleep parameters according to the sensitivity-threshold settings of actigraphy (automatic, high, medium, and low) and also to compare sensitivity, specificity, PVS, PVW, and kappa of the epoch-by-epoch agreement analysis, for each sensitivity settings.

In order to make pairwise comparisons of all ACT sensitivity-threshold settings for each sleep parameter, we computed a *post hoc* test, the Tukey’s range test.

## Results

### Study Participants

Among the 29 subjects who initiated the combined recording of actigraphy and PSG, one participant had incomplete data because he pulled the PSG electrodes off during the night, one participant did not tolerate the actigraph, and for one participant, a technical issue with the actigraph compromised the data. Overall, 26 participants completed the concurrent PSG and ACT recordings and were included in the final analysis. The sample included 20 boys and six girls, with a mean age of 5.36 ± 1.57 years and an age range [2.94–8.1] years.

As seen in **Table 2**, most of the children exhibited a developmental delay in adaptive behaviors with delays ranging from [0.58–5.92] years.

**Table 2:** Descriptive characteristics of the study participants.

Population description (N=26)	% (N)	Mean (SD)	Range
Demographic characteristics			
Gender (boys)	77% (20)	
Chronological age (years)			5.36 (1.57)	[2.94–8.10]
VABS subscale equivalent age			
Daily living skills (years)		2.66 (1.19)	[1.25–5.00]
Communication (years)		2.32 (1.34)	[0.75–5.83]
Motor skills (years)		3.21 (1.45)	[1.67–5.92]
Socialization (years)		1.98 (1.03)	[0.58–4.00]

VABS, Vineland Adaptive Behavior Scales (34).

### Actigraphy Sensitivity-Threshold Setting

The low sensitivity-threshold setting of actigraphy showed the best fit for the comparison of actigraphy-derived sleep parameters to PSG and was thus reported here. Results for other settings (low, medium, high, automatic) can be found in **Supplementary Data**.

### Sleep Parameter Agreement Analyses

#### Intraclass Correlation Coefficient

ICC highlighted a good correlation between PSG and ACT for SL and TST, and a moderate agreement for WASO and SE (**Table 3**).

**Table 3:** Intraclass correlations between actigraphy and polysomnography.

Sleep parameters	ICC
SL	0.795
WASO	0.731
TST	0.850
SE	0.689

ICC ranges from 0 (no agreement) to 1 (perfect agreement). ICC < 0.5 indicates poor agreement, 0.5 < ICC > 0.75 indicates moderate agreement, 0.75 < ICC > 0.9 indicates good agreement, and ICC > 0.90 indicates perfect agreement (29). ICC, Intraclass correlation coefficient; SL, sleep latency; WASO, wake after sleep onset; TST, total sleep time; SE, sleep efficiency.

#### Bland-Altman Plots

As a reminder (see the section *Sleep Parameters Agreement Analyses*), in this method, the differences between the two techniques (i.e., SL according to actigraphy minus SL according to PSG) are plotted against their averages. Differences are expressed as PSG - actigraphy, so a negative value indicates actigrahy overestimated the sleep parameter, whereas a positive value indicates actigraphy underestimated the sleep parameter.

**Figure 1:**
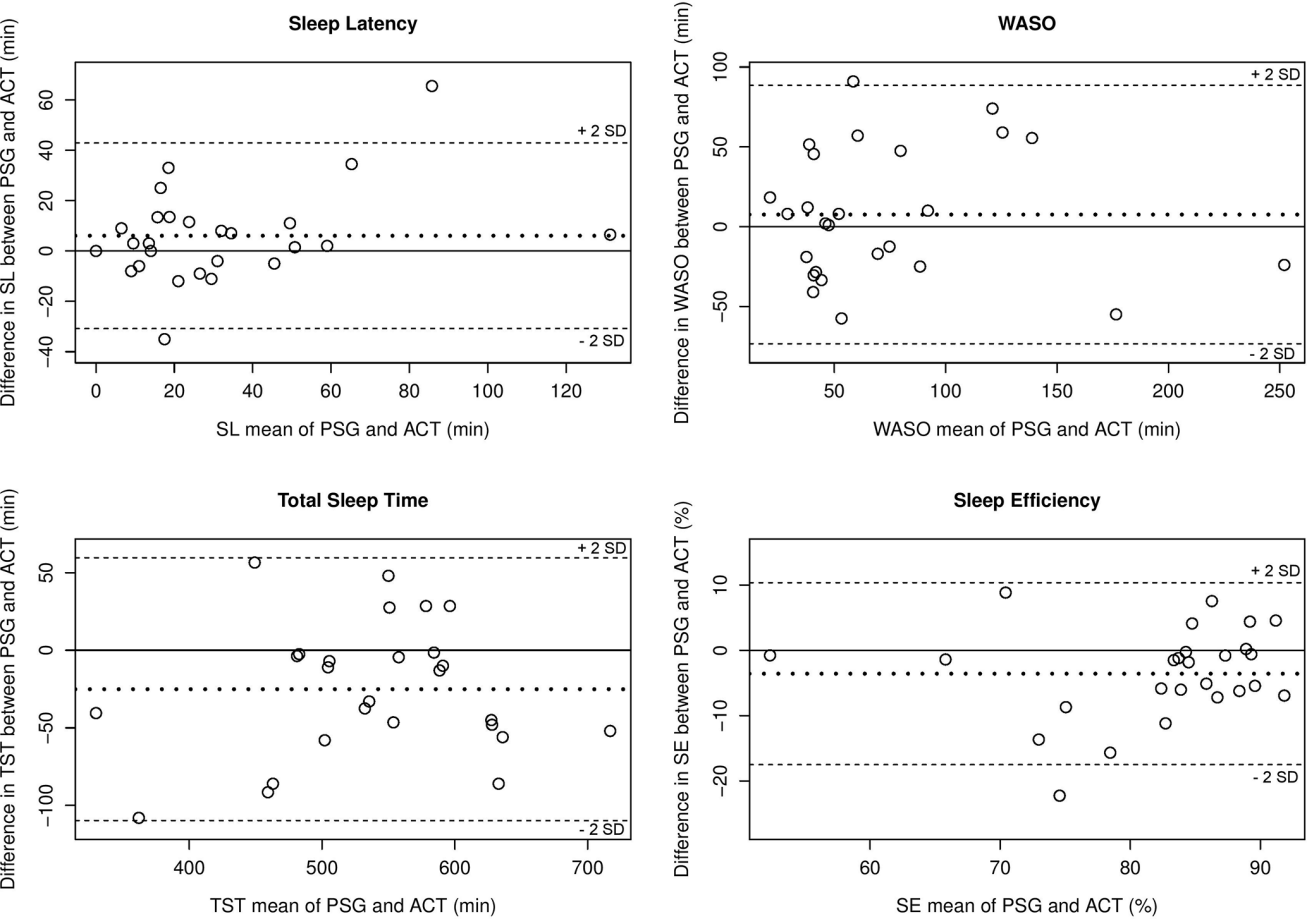
Bland-Altman plots (35) for the comparison of PSG and ACT for each sleep parameter. The mean of each sleep parameter with the two techniques is represented in the *x*-axis and differences (i.e., mean biases) for each sleep parameter between the two techniques are represented in the *y*-axis. Each subject is represented by a dot. The continuous line which passes through zero, representing perfect agreement between PSG and ACT, is the reference line. The bold dotted line represents the mean difference of the study sample (i.e., mean bias) for each sleep parameter with the two techniques. Differences are expressed as PSG—actigraphy, so a negative value indicates actigraphy overestimated the sleep parameter, whereas a positive value indicates actigraphy underestimated the sleep parameter. SL, sleep latency; WASO, wake after sleep onset; TST, total sleep time; SE, sleep efficiency.

The Bland-Altman plots revealed that, for almost all participants, the differences between ACT and PSG fall between the [-2SD; + 2SD] limits of agreement (**Figure 1**). In average, ACT underestimates SL (mean difference = 6.06 min) and WASO (mean difference = 7.57 min) and overestimates TST (mean difference = -25.09 min) and SE (mean difference = -3.58%).

#### Equivalence Tests

**Figure 2:**
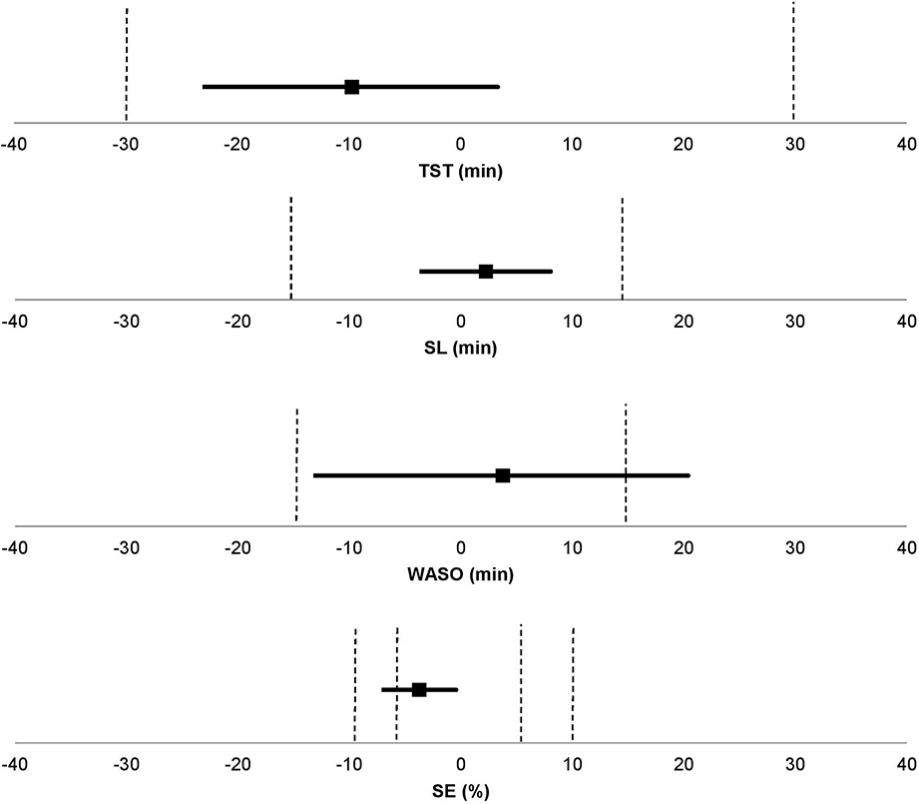
Equivalence tests (28) between PSG and ACT for each sleep parameter. The pre-set ranges of acceptability (represented by the dashed lines) were set to ± 30 min for TST, ± 15 min for SL and WASO, and ± 5% and ± 10% for SE. SL, sleep latency; WASO, wake after sleep onset; TST, total sleep time; SE, sleep efficiency.

The equivalence tests allow to conclude if the two techniques are *clinically* equivalent within a pre-set range of acceptability, and a p-value inferior to 0.05 indicating equivalence (as detailed in the section *Sleep Parameter Agreement Analysis*). A clinical equivalence between ACT and PSG was observed within the pre-set range of acceptability for SL (p < 0.001) and TST (p<0.01) (**Figure 2**). SE was equivalent in the two methods when using the less conservative range of acceptability of 10% (p < 0.01) but not when using the conservative range of 5% (p = 0.25). WASO measured by ACT was not equivalent to PSG (p = 0.13).

### Epoch-by-Epoch Agreement Analysis

Sensitivity, specificity, accuracy, PVS, PVW values, and Cohen’s kappa coefficient of epoch-by-epoch comparisons between ACT and PSG are shown in **Table 4**. ACT showed high sensitivity and moderate specificity in classifying epochs into sleep or wake. The accuracy between ACT and PSG was high, and the kappa values showed substantial agreement. PVS was high, whereas PVW was moderate.

**Table 4:** Epoch-by-epoch agreement analysis between ACT and PSG.

Epoch-by-epoch agreement indicators	(Mean ± SD)
Sensitivity	0.939 ± 0.057
Specificity	0.511 ± 0.201
Accuracy	0.868 ± 0.077
PVS	0.901 ± 0.076
PVW	0.640 ± 0.166
Kappa	0.735 ± 0.154

Agreement indicators are defined in the methods section (Epoch-by-Epoch Agreement Analysis). Kappa coefficient between 0–0,2 = slight agreement; 0,2–0,4 = fair agreement; 0,4–0,6 = moderate agreement; 0,6–0,8 = substantial agreement; 0,8–1,0= almost perfect agreement (33). PVS, predicted value for sleep; PVW, predicted value for wakefulness.

## Discussion

To the best of our knowledge, this is the first study to demonstrate the validity of actigraphy for the assessment of sleep in children with ASD, compared to polysomnography, the gold standard. The comparison of PSG and ACT included two sets of state-of-the-art comparisons and analyses: the agreement analysis of the four sleep parameters and an epoch-by-epoch agreement analysis.

Sleep parameter agreement analyses revealed, on the basis of ICC, a good agreement between PSG and ACT for the assessment of SL and TST, and a moderate agreement for WASO and SE. Similarly, we observed a clinically satisfactory equivalence for the measurement of SL and TST but not for WASO between ACT and PSG, within the pre-set range of acceptable deviation. SE was significantly equivalent in the two methods when using the less conservative range of acceptability of 10% (p < 0.01) but not when using the conservative range of 5% (p = 0.25). The Bland-Altman plots revealed that, for almost all participants, the differences between ACT and PSG fell between [-2SD; +2SD] limits of agreement, and there were no visible trends indicating that ACT performed differently with respect to SL, TST, WASO, or SE values.

Epoch-by-epoch agreement analyses showed high sensitivity, PVS and accuracy, substantial kappa, but moderate PVW and specificity. These results indicate that ACT has a high ability to identify TS, but lower ability to detect TW. According to Sadeh’s recommendation, one should look for a specificity higher than 0.60, in the epoch-by-epoch ACT/PSG comparisons (36). In our study, we found a moderate specificity of 0.51 with the low sensitivity-threshold setting. With the medium and high sensitivity-threshold settings (results in **Supplementary, Table S3**), we obtained better specificity (respectively, 0.617 ± 0.193 and 0.699 ± 0.18), but all other indicators were lower (specificity and PVW) or equivalent (accuracy, kappa, and PVS), and above all, sleep parameters (TST, WASO, and SE) were less accurate with those settings (results in **Supplementary, Table S3**).

To date, most studies which have investigated the validity of actigraphy, by comparing it to polysomnography, were conducted in healthy young adults (25, 32, 37). However, actigraphy is a particularly useful tool in pediatric research and thus widely employed, even though some devices have not been validated yet in this population. Actigraphy has been validated in the TD pediatric population, e.g., in infants and young children (38–40) and also in adolescents (41). Although results of previous studies may be misleading as different devices and scoring algorithms do not perform equally across age groups (24), the agreement indicators of the present study were within the range of those reported in the literature ([0.88–0.93] for the sensitivity, [0.46–0.77] for the specificity, and [0.84–0.9] for the accuracy) (24, 42). Thus, our results are consistent with what has been reported in the TD pediatric population: actigraphy correctly identifies sleep periods (as denoted by the high sensitivity) but is less accurate in identifying WASO (as denoted by the low specificity). Despite the growing interest in actigraphy research, validation studies in children are still lacking. This is of particular importance as children display different sleep behaviors than adults with children displaying more movements during sleep than adults. Validation studies should take into consideration these differences and examine sleep across different developmental age groups.

Within the pediatric population, there are children for whom sleep disorders are much more prevalent. Indeed, up to 80% of young children with ASD have sleep disorders (43–45) compared to about 25% of TD children (46). These disturbances among the ASD population are three to four times more common and have been related to daytime symptomatology (12–15), hence the importance of measuring sleep in this population.

The vast majority of sleep studies in the ASD population have focused on subjective assessment of sleep (i.e., parent-reported sleep questionnaires or sleep diaries) because of the difficulties performing more objective assessments (e.g., actigraphy, polysomnography). Indeed, in 2015, according to Elrod and Hood’s meta-analysis (7), there were only 10 studies that examined sleep objectively using PSG (11, 47, 48) and/or ACT (49–51). The PSG sleep parameters observed in our study were similar to those described by Buckley et al. (52), who included 60 ASD children aged 2.24–13.1 years. In the present study, compared to theirs, were reported, respectively: a SL of 35.2 min (±33.2) against 39.4 min (±33.2), a WASO of 77.2 min (±56) against 73.5 min (±72.2), and a SE of 80.3% (±10.1) against 80.2% (±12.7). In average, TST was 47 min higher in our sample compared to Buckley et al. (52), but the SD were very large on this measure for both studies (respectively, 91 and 126 min). For actigraphy, results are too device and setting-dependent to allow a direct comparison between studies. For example, in the current literature, reported WASO range from 18 to 88 min (50, 51) (for details see Methodology and **Supplementary Data**). As many children with ASD present sensory abnormalities, and thus may not tolerate electrodes on their scalp or face, PSG recording may be compromised. Actigraphy has become more and more popular as an alternative, non-invasive, objective, and cost-effective assessment tool for the study of rest-activity cycles as a proxy to sleep–wake rhythms. It has become a tool of choice for the assessment of sleep quality in children with ASD but has not been validated yet in this population. Our study provides, for the first time, the mandatory evidence for the validity of actigraphy compared to PSG for these assessments in children with ASD, providing thus the basis for improved medical care of sleep disorders in these children. It is essential to validate sleep measures in other specific pediatric populations, as results found in the TD children cannot be extrapolated to them.

As reported by Meltzer et al. (53), it is common to find validation-type studies using inadequate methodology and relying only on correlation analyses, or alternatively focusing more on sensitivity than specificity, leading to deceptive results. To address this, we used multiple comparison and performed recommended agreement analysis methods (27, 28). We also compared both clinical sleep parameters and recorded epochs, to report sensitivity and specificity. Moreover, in the literature, results regarding actigraphy-derived sleep quality parameters in children with ASD are contradictory (10, 50). This may be due to variable wake sensitivity threshold that many studies do not report. Thus, we compared several sensitivity-threshold settings.

A particular strength of the present study is that we included ASD children across the spectrum, with and without associated intellectual disability ranging from low to high severity (with respect to DSM-5). This allows to generalize our results to the overall population of ASD children and not only to high functioning ASD children, as is often the case. In our study, we find the same male predominance in ASD population [i.e., six girls and 20 boys (54)]. Our study sample was young, with a mean age of 5.36 years. It is important to study sleep quality in children and detect sleep difficulties as early as possible, especially since untreated sleep difficulties maintain with time and become chronic.

Despite these strengths, there are also some limitations to this study. The main limitation is the relatively small sample size of 26 subjects and high interindividual variability, which may both reduce statistical power, especially regarding equivalence tests. However, the sample size was above the average sample size of previous studies examining the validity of actigraphy against polysomnography reported by Meltzer et al. (53), which was of 18 (range [8–45] participants). Also, the interindividual variability of the present study is similar to that reported in previous studies (11, 14, 55) and did not refrain from finding that actigraphy is a valid method to assess sleep quality parameters in children with ASD, compared to polysomnography. Furthermore, we have included children within the broad spectrum of ASD and did not only focus on a homogeneous group of high functioning children with ASD, thus reflecting a more representative population of children with ASD and sleep disturbances such as those seen in sleep clinics—which is a strength of this study. We also compared only a single night of actigraphy to concurrent PSG recording while it is recommended to collect five to seven nights of recording across devices (56). This can be explained by the fact that it is not feasible to carry-out seven consecutive days of polysomnography in children with ASD, because of sensory abnormalities or associated behavior disturbances. Lastly, it should be noted that the data sampling over the actigraph recording was set to 1-min epochs and retrospectively converted to 30-s epochs by the software to allow comparison with the PSG epoch settings, as done by previous studies (24, 25). Although this has little influence on the comparison of general sleep parameters, it can lead to a small artificial reduction of epoch-by-epoch agreement indicators, including specificity and sensitivity. For a better comparability between PSG and ACT epochs, future studies should insure automated synchrony in timing and epoch lengths.

In addition to these limitations, it is important for researchers and clinicians to understand that sleep/wake scoring depends on specific devices, algorithms, and sensitivity thresholds. The default mode of an actigraph may be valid in one specific population but not in another. For scoring purposes, a daily diary and/or event marker use, when possible, is necessary in order to accurately identify sleep periods.

## Conclusion

To our knowledge, our study is the first to validate actigraphy as a method to assess sleep quality parameters compared to polysomnography, using a particularly sound state-of-the-art methodology, in a young population of children with ASD. The results suggest actigraphy to be a valid method to evaluate sleep within a particularly vulnerable population, with a high sensibility and a good reliability for most sleep parameters, including TST and sleep onset latency. With the increasing number of research studies using actigraphy, it is important to have multiple validation studies for each device and each developmental age, across healthy and clinical samples. This study confirms ACT as the objective alternative to assess sleep quality in children with ASD and can provide reliable information to clinicians when investigating sleep to improve quality of life.

## Data Availability

The datasets generated for this study are available on request to the corresponding author.

## Ethics Statement

This study was carried out in accordance with the recommendations of the regional French Institutional Review Board with written informed consent from parents of participants and assent was obtained from the child when possible. All parents of subjects gave written informed consent in accordance with the Declaration of Helsinki. The protocol was approved by the regional French Institutional Review Board (CPP Est IV 11/04/2012).

## Author Contributions

EY-K collected the data, conducted the actigraphy and polysomnography data processing, and wrote the manuscript. ER planned and performed the analyses and contributed to manuscript revision. M-MG collected part of the data and assisted with manuscript review and revisions. NL collected part of the actigraphy and polysomnography data and assisted with manuscript review and revisions. PF collected part of the actigraphy and polysomnography data and assisted with manuscript review and revisions. PB assisted with the study design and the funding and contributed to manuscript review and revisions. CS designed the study, secured funding, collected the data, assisted with data interpretation and critique, as well as contributed to manuscript review and revisions. All authors are responsible for the reported research and have reviewed and approved the final manuscript as submitted.

## Funding

This work was supported by a grant from the National Hospital Program for Clinical Research (PHRC National Autisme et Sommeil, HUS N°5060, 2012). ER received a grant from SFRMS and EY-K from Ensar Foundation.

## Conflict of Interest Statement

The authors declare that the research was conducted in the absence of any commercial or financial relationships that could be construed as a potential conflict of interest.
