# Supplementary material for: Validity of Actigraphy Compared to Polysomnography for Sleep Assessment in Children With Autism Spectrum Disorder
Source: Front Psychiatry. 2019 Aug 2;10:551. doi: 10.3389/fpsyt.2019.00551 (PMC6688709; doi:10.3389/fpsyt.2019.00551)
Supplement: Supplementary file 1 [file DataSheet_1.pdf]

Supplementary data – Comparison of actigraphy sensitivity-threshold settings

In the present supplementary data, results of comparison across different actigraphy sensitivity-threshold settings are reported. There are 4 sensitivity-threshold settings: the low sensitivity-threshold is defined with a threshold level of 80 counts. It means that an activity score in an epoch of 80 counts or more is sufficient for that epoch to be scored as awake. The medium sensitivity-threshold corresponds to a threshold level of 40 counts, the high sensitivity-threshold to a threshold level of 20 counts and the automatic sensitivity-threshold to a variable threshold level. For the latter, the software derives from the subject's activity level a subject-adapted threshold.

These thresholds significantly affect the scoring of epochs into 'sleep' versus 'wake', as well as the identification of wake during the night; the sleep start and sleep end, however, are calculated independently. The following results will thus compare the epoch-by-epoch agreement analysis values (i.e., sensitivity, specificity, accuracy, Cohen's kappa coefficient, Predicted Value for Sleep (PVS) and Predictive Value for Wake (PVW)) and the WASO sleep parameter across actigraphy sensitivity-threshold settings.

WASO distribution across actigraphy sensitivity-threshold settings

The WASO values across the sensitivity settings, as presented in Figure S1, were significantly different (ANOVA p-value <0.001). Automatic and low-sensitivity settings showed the best fit for the comparison of actigraphy-derived WASO and PSG with average differences of respectively -4.5 $CI_{95\%}$ [-11.4 to 2.3] and -3.6 $CI_{95\%}$ [-10.4 to 3.3]. Tukey's post-hoc test showed significant differences between the ACT high-sensitivity setting and both the ACT automatic ($p<0.001$) and low ($p=0.003$) sensitivity settings. The difference between the medium-sensitivity setting and the other settings was not significant (medium vs automatic $p=0.16$, medium vs low $p=0.33$).

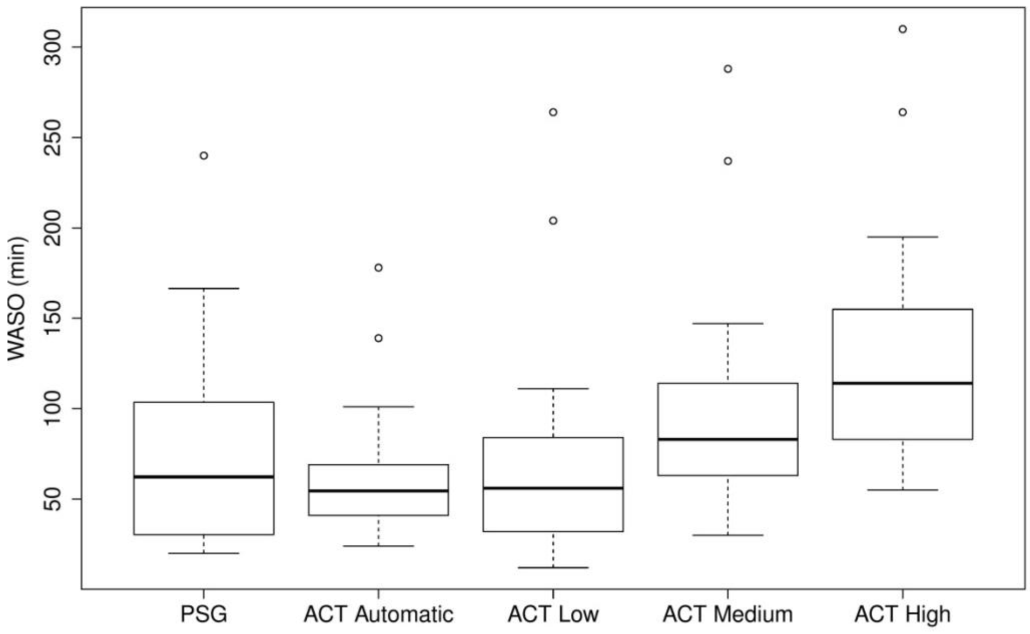

Figure S1. Boxplots of wake after sleep onset (WASO) across sensitivity-threshold settings, compared to polysomnography (PSG)

ICC comparison across actigraphy sensitivity-threshold settings

ICC ranges from zero (no agreement) to 1 (perfect agreement). An ICC < 0.5 indicates poor agreement, 0.5 < ICC > 0.75 indicates moderate agreement, 0.75 < ICC > 0.9 indicates good agreement and ICC > 0.90 indicates perfect agreement (Koo and Li, 2016).

ICC (Watson and Petrie, 2010) highlighted a moderate agreement for WASO across sensitivity-threshold settings (automatic setting, ICC = 0.653; high setting, ICC = 0.563; medium setting, ICC = 0.729; low setting, ICC = 0.731).

Bland-Altman plots comparison across actigraphy sensitivity-threshold settings

In this method, the differences between the two techniques (i.e., WASO according to actigraphy minus WASO according to PSG) are plotted against their average (Bland and Altman, 1999).

In average, with the automatic sensitivity-threshold setting, ACT underestimated WASO (mean difference = 14.15 minutes). With high sensitivity-threshold setting, ACT overestimated WASO (mean difference = -130.53 minutes). With medium and low sensitivity-threshold settings, ACT underestimated WASO with a mean difference of -19.39 minutes and 7.57 minutes respectively.

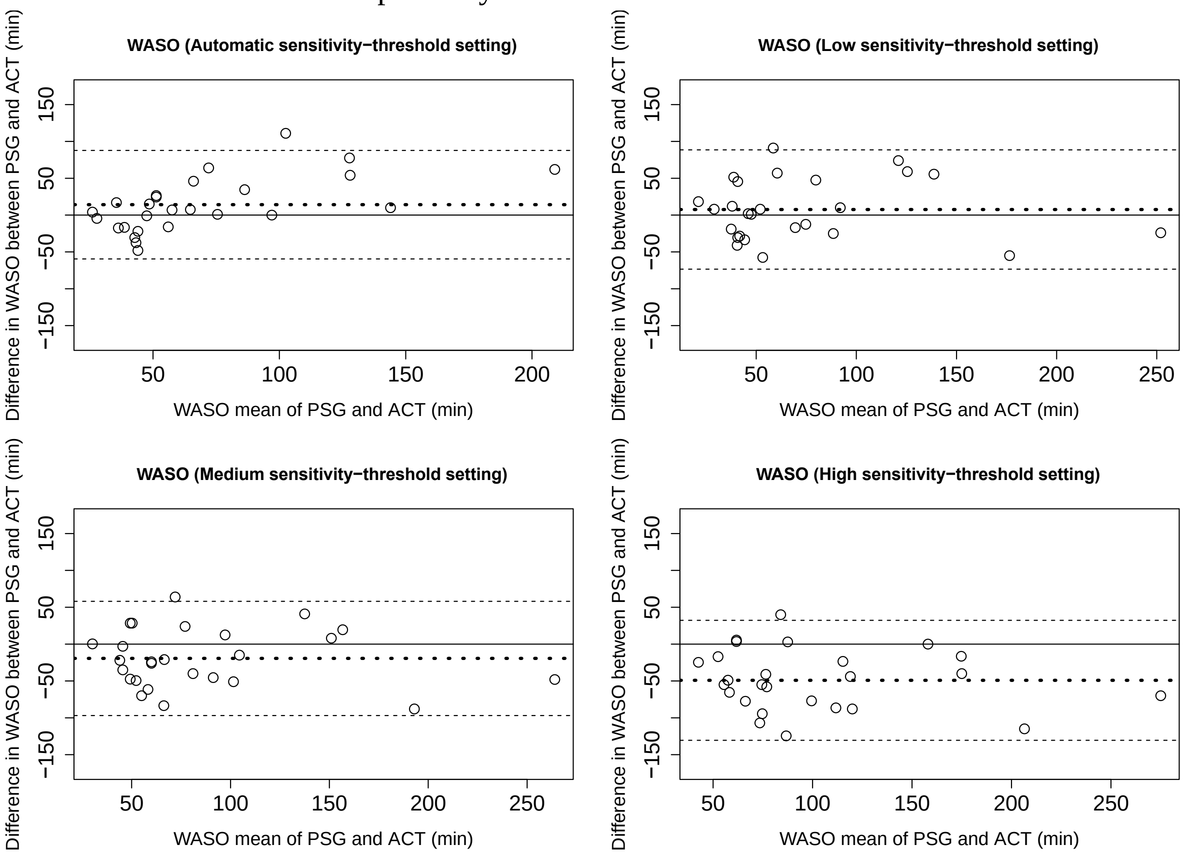

Figure S2. Bland-Altman plots based comparison between actigraphy and polysomnography
The mean of WASO with the two techniques is represented in the *x*-axis and differences (i.e., mean biases) for WASO between the two techniques are represented in the *y*-axis. Each subject is represented by a dot.

The continuous line which passes through zero represents perfect agreement between PSG and ACT, it is the reference line.

The bold dotted line represents the mean difference of the study sample (i.e., mean bias) for WASO with the two techniques.

Differences are expressed as PSG – actigraphy, so a negative value indicates actigraphy overestimated the sleep parameter, whereas a positive value indicates actigraphy underestimated the sleep parameter.

WASO, wake after sleep onset.

Test for equivalence comparison across actigraphy sensitivity-threshold settings
(Wieringen and Cribbie, 2014)

Within the pre-set range of acceptability, established to [-15 min; +15 min], WASO measured by ACT was not equivalent to PSG in any sensitivity-threshold setting according to Yuen two one-sided paired tests for equivalence (p>0.05) (Figure S3).

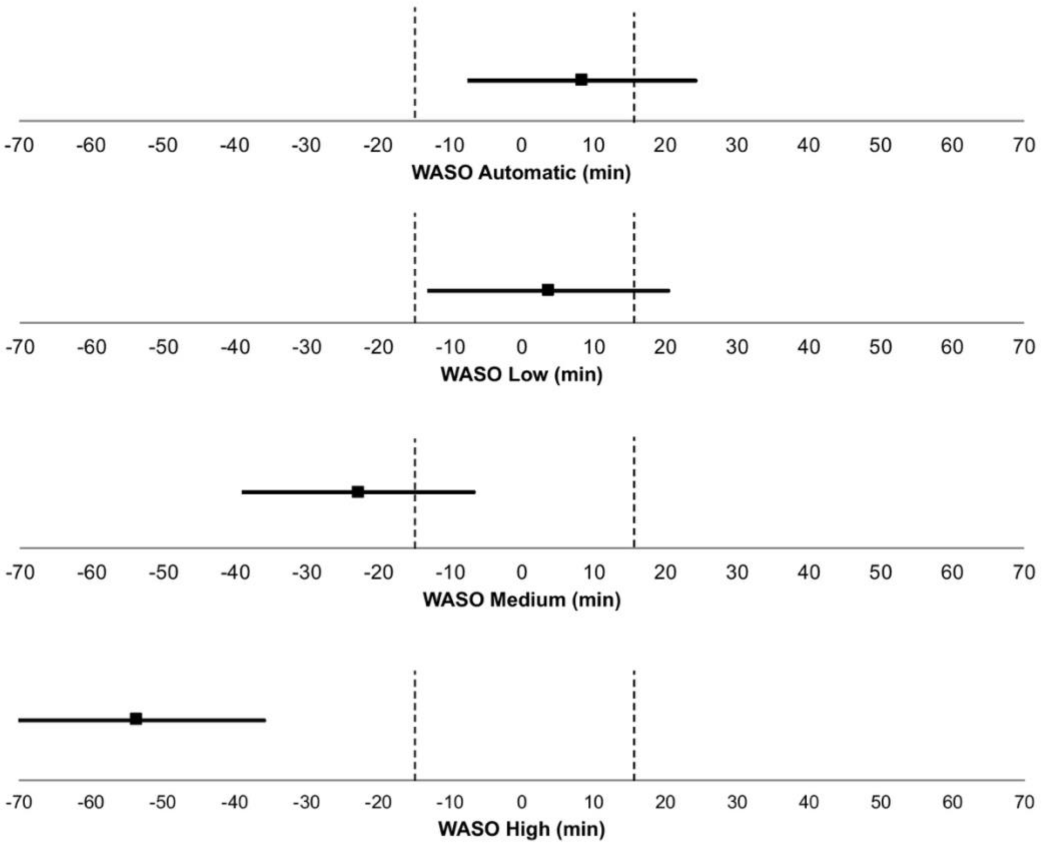

Figure S3. Equivalence tests between PSG and ACT for WASO
The ranges (represented by the dashed lines) were set to ± 15 minutes for WASO.
WASO, wake after sleep onset

Epoch-by-Epoch comparison across actigraphy sensitivity-threshold settings

Sensitivity, specificity, accuracy, Cohen's kappa coefficient, PVS and PVW values of epoch-by-epoch comparisons between ACT and PSG are shown in Table S1. ACT showed high sensitivity and moderate specificity for each setting. The accuracy between ACT and PSG was high for each sensitivity-threshold setting. Kappa values showed moderate (0.67 for high sensitivity) to substantial agreement (from 0.71 to 0.74 for automatic, medium and low sensitivity). PVS were high whereas PVW were moderate. Three of the agreement indicators varied across ACT settings: the sensitivity ($p<0.001$), the specificity ($p<0.001$) and the PVW ($p=0.008$). Tukey's post-hoc analyses showed significant differences between the high and the low sensitivity-threshold settings for sensitivity ($p<0.001$), specificity ($p=0.002$) and PVW ($p=0.016$), all other associations were non-significant.

Table S1. Epoch-by-epoch agreement analysis

	**ACT by actigraphy sensitivity-threshold settings (mean ± SD)**				
**Agreement indicators**	**Low**	**Medium**	**High**	**Auto**	**p value**
Sensitivity	0.94 ± 0.06	0.90 ± 0.06	0.86 ± 0.07	0.94 ± 0.05	**<0.001**
Specificity	0.51 ± 0.20	0.62 ± 0.19	0.67 ± 0.18	0.51 ± 0.15	**<0.001**
Accuracy	0.87 ± 0.08	0.86 ± 0.07	0.83 ± 0.07	0.86 ± 0.08	0.359
PVS	0.90 ± 0.08	0.92 ± 0.07	0.93 ± 0.07	0.90 ± 0.08	0.366
PVW	0.64 ± 0.17	0.56 ± 0.15	0.50 ± 0.15	0.63 ± 0.19	**0.008**
Kappa	0.74 ± 0.15	0.71 ± 0.14	0.67 ± 0.14	0.73 ± 0.16	0.364

p value (ANOVA) for the comparison of all actigraphy settings.

PVS, predicted value for sleep; PVW; predicted value for wakefulness; Auto, Automatic actigraphy settings; High-medium-low, high-medium-low actigraphy settings.
